# Application of Detergents or High Hydrostatic Pressure as Decellularization Processes in Uterine Tissues and Their Subsequent Effects on *In Vivo* Uterine Regeneration in Murine Models

**DOI:** 10.1371/journal.pone.0103201

**Published:** 2014-07-24

**Authors:** Erna G. Santoso, Keita Yoshida, Yasushi Hirota, Masanori Aizawa, Osamu Yoshino, Akio Kishida, Yutaka Osuga, Shigeru Saito, Takashi Ushida, Katsuko S. Furukawa

**Affiliations:** 1 Department of Mechanical Engineering, School of Engineering, The University of Tokyo, Tokyo, Japan; 2 Department of Obstetrics and Gynecology, School of Medicine, The University of Tokyo, Tokyo, Japan; 3 Department of Obstetrics and Gynecology, Faculty of Medicine, University of Toyama, Toyama, Japan; 4 Department of Material-based Medical Engineering, Division of Biofunctional Restoration, Institute of Biomaterials and Bioengineering, Tokyo Medical and Dental University, Tokyo, Japan; 5 Department of Bioengineering, School of Engineering, The University of Tokyo, Tokyo, Japan; Institute for Frontier Medical Sciences, Kyoto University, Japan

## Abstract

Infertility caused by ovarian or tubal problems can be treated using In Vitro Fertilization and Embryo Transfer (IVF-ET); however, this is not possible for women with uterine loss and malformations that require uterine reconstruction for the treatment of their infertility. In this study, we are the first to report the usefulness of decellularized matrices as a scaffold for uterine reconstruction. Uterine tissues were extracted from Sprague Dawley (SD) rats and decellularized using either sodium dodecyl sulfate (SDS) or high hydrostatic pressure (HHP) at optimized conditions. Histological staining and quantitative analysis showed that both SDS and HHP methods effectively removed cells from the tissues with, specifically, a significant reduction of DNA contents for HHP constructs. HHP constructs highly retained the collagen content, the main component of extracellular matrices in uterine tissue, compared to SDS constructs and had similar content levels of collagen to the native tissue. The mechanical strength of the HHP constructs was similar to that of the native tissue, while that of the SDS constructs was significantly elevated. Transmission electron microscop*y* (TEM) revealed no apparent denaturation of collagen fibers in the HHP constructs compared to the SDS constructs. Transplantation of the decellularized tissues into rat uteri revealed the successful regeneration of the uterine tissues with a 3-layer structure 30 days after the transplantation. Moreover, a lot of epithelial gland tissue and Ki67 positive cells were detected. Immunohistochemical analyses showed that the regenerated tissues have a normal response to ovarian hormone for pregnancy. The subsequent pregnancy test after 30 days transplantation revealed successful pregnancy for both the SDS and HHP groups. These findings indicate that the decellularized matrix from the uterine tissue can be a potential scaffold for uterine regeneration.

## Introduction

Infertility is often associated with the inability of women to either conceive or maintain pregnancy. On average worldwide, 72.4 million or 9% of women at the reproductive age are infertile [1]. The infertility can be attributed to various reasons such as: ovulatory dysfunction [2], [3], tubal diseases [4], [5], endometriosis [6]–[8] and uterine malformations [9], [10]. A common infertility treatment is assisted reproductive technology (ART), which can work for ovulatory and tubal disorders but not for uterine abnormalities. In addition, gynecological malignancies such as cervical and endometrial cancers often lead to the removal of the uterus by hysterectomy, which prevents the women from carrying out future pregnancies.

A current solution for this type of infertility includes gestational surrogacy [11]. Gestational surrogacy is often preferred as it carries the lowest risk and highest success rate. However, it is impossible to prevent the possibility of accidents such as complications and death of the surrogate mother during childbirth. The conception and childbirth impose physical and mental stresses on the surrogate mother. In some cases, a suitable surrogate mother might not be found for the couple. Moreover, by using surrogacy, the intended mother would not be able to experience the childbearing process.

For women who prefer to carry out the pregnancy themselves, uterus transplantation [12] has gotten a lot of attention. Recently, the success rate of uterine transplantation has been steadily increasing, and one study also showed the possibility of embryo implantation following surgery [13], [14]. However, to date, there is still no report of childbirth following a uterine transplantation in a human. Furthermore, due to ethical reasons and risks for donors and/or recipients, uterus transplantation from a living donor is restricted on a case-by-case basis [15], [16], while extracting a uterus from a dead donor has a higher risk of incompatibility [17]. In all cases, following transplantation recipients must be treated with immunosuppressive drugs for a lifetime.

In these circumstances, uterus reconstruction by tissue engineering approach is considered an attractive approach. Biological materials, such as collagen [18], for uterine tissue regeneration has been reported. While promising, the regeneration speed was considered slow. On top of this, due to the nature of collagen scaffold, the tissue regeneration is dependent on existing native tissue, making it inapplicable to whole uterine tissue engineering. Considering these obstacles, the application of decellularized matrices as scaffold for uterine tissue engineering was taken as the focus of this study.

Decellularized matrices have been reported for scaffolding in many vital organs such as the kidney [19], [20], heart [21], [22], blood vessel [23], [24], and bone [25], [26]. In decellularization, the tissue of interest is subjected to extreme conditions such as high acidity, alkalinity or pressure. With these methods, disruption of the cell membrane occurs and causes cell death. Removal of the dead cells without altering the overall properties of tissue can be done using a washing buffer containing enzymes. Due to the preservation of original tissue properties, a lower immune response for the host has been reported [27]. Consequently, we hypothesized that this method is highly useful for the purpose of uterus reconstruction and is under-explored.

In this study, we first assessed three different decellularization methods for their applicability to uterine tissues, namely sodium dodecyl sulphate (SDS), high hydrostatic pressure (HHP) and Triton-X. Through this, we created scaffold with high bio- and structure- compatibility to the native uterus. Subsequently, an in vivo study using decellularized tissue was performed and evaluated for the capability of reconstruction and the responsiveness to the ovarian hormone. In addition, the capability for pregnancy was investigated in order to confirm the possibility of utilizing decellularized uterine tissues as scaffolds in uterine reconstruction.

## Materials and Methods

### Uterine tissue sample preparation

All animals used in this investigation were housed in the University of Tokyo Animal Care Facility according to the institutional guidelines for the use of laboratory animals. The experimental procedures were approved by the institutional animal experiment committee. The title of the approved animal experiment plan is “Rat uterine tissue engineering (ID number is P12-113; Duration is January 8, 2012 to January 7, 2017; responsible person is Yutaka Osuga)”, which was finally approved by the Animal Experimentation Committee, Faculty of Medicine, University of Tokyo, as of December 16, 2011. Sprague Dawley rats (SD rats, female, 9 weeks old) were purchased from CLEA Inc. Japan. We put down the rats painlessly under the overdose of anesthelic agent, isoflurane (Mylan, USA). Uterine horns were excised from rats having a proestrus and metestrus cycle and trimmed of connective tissue and fat. Briefly, the horns were rinsed of blood with phosphate buffered saline (PBS), incise in the mesometrium line and cut into 15 mm×5 mm rectangular samples.

### Decellularization of uterine tissue

#### Ionic detergent (Sodium dodecyl sulfate; SDS) treatment

SDS was made according to the method by Booth et al. [28] with a slight modification. Briefly, SDS (Wako, Japan) was dissolved in PBS and sterilized. Up to four samples at a time from the same rat were immersed in 5 ml of SDS solution at room temperature with the following conditions: 0.1% SDS/PBS for 1 hour, 1% SDS/PBS for 1 hour, or 1% SDS/PBS for 2 hours.

After SDS treatment, samples were washed for 1 week at 4°C using a washing buffer containing 0.9% NaCl (Wako, Japan), 0.05 M magnesium chloride hexahydrate (Wako, Japan), 0.2 mg/ml DNAse I (Roche, USA) and 1% penicillin and streptomycin (Gibco, Japan) on a shaker set at frequency of 1 Hz.

#### High hydrostatic pressure (HHP) treatment

Decellularization by HHP was achieved by using a cold isostatic pressurization machine (Dr. Chef; Kobelco, Japan) according to the method by Funamoto et al. [29] with adjustment. Samples from the same rat were packed together in a polyethylene bag filled with saline solution and immersed in transmission fluid in the sample chamber of the machine. The onset temperature was set at 10°C or 30°C. The chamber was pressurized to 980 MPa at 196.1 MPa/min for an onset temperature of 10°C and 65.3 MPa/min for an onset temperature of 30°C, and then held for 10 minutes before being reduced back to atmospheric pressure at 196.1 MPa/min or 65.3 MPa/min for the onset temperatures of 10°C and 30°C, respectively. After decellularization, samples were washed for 1 week at either 4°C or 37°C in a washing buffer, as previously described in SDS treatment, on a shaker set at a frequency of 1 Hz.

#### Non-ionic detergent (Triton X-100)

Triton X-100 solution was made according to the method by Bader et al. [30] with modification. Triton X-100 (Sigma-Aldrich, Japan) was dissolved directly in PBS and sterilized. Up to four samples at a time from the same rat were immersed in 5 ml of Triton X-100 solution at room temperature with the following conditions: 1% Triton X-100/PBS for 24 hour, 3% Triton X-100/PBS for 24 hour, or 3% Triton X-100/PBS for 48 hours. After decellularization, samples were washed for 1 week at 4°C in a washing buffer, as previously described in the SDS treatment, on a shaker set at a frequency of 1 Hz.

### Histology

Uterine tissues were briefly rinsed with PBS containing 1% penicillin and streptomycin and fixed in 10% neutral buffered formalin solution. Next, samples were dehydrated in graded alcohol, embedded in paraffin blocks and sectioned at 4 µm thickness. Samples were stained with Hematoxylin & Eosin (nucleus and cytoplasm), Masson’s Trichrome (collagen) and Verhoeff’s Van Gieson (elastin). Samples were observed using an optical microscope (Zeiss, Germany) equipped with camera.

Implants were stained with vimentin for stroma cells (Abcam plc, UK; diluted 1∶200), smooth muscle alpha actin (α-SMA) for smooth muscle cells (Abcam plc, UK; diluted 1∶400), Ki67 for proliferating cells (Abcam plc, UK; diluted 1∶100), CD31 for endothelial cells (Santa Cruz Biotechnology, Inc., USA; diluted 1∶100) and estrogen receptor (ER) (Abcam plc, UK; diluted 1∶200). Heat induced epitope retrieval (10 mM citrate buffer, pH 6.0) was performed for Ki67, CD31 and ER prior to endogeneous peroxidase inactivation. Primary antibody incubation was performed in 4°C overnight followed by secondary antibody (goat-anti-rabbit IgG; Santa Cruz Biotechnology, Inc., USA; diluted 1∶200) incubation for 1 hour at room temperature. Afterwards, samples were stained with 3,3′ Diaminobenzidine (DAB) and counterstained with hematoxylin.

### Mechanical test

Extracted uterine horns were cut into ring-shaped samples with a length of 3–6 mm and then opened up into a rectangular shape. Mechanical tests were carried out using an autograph AGS-5kNG (Shimadzu, Japan). The thickness was determined as the point where the load cell detected a compressive force of 0.02 N when the sample was positioned flat on the stage and loaded in compression. Next, a uniaxial tensile test was done on each of the samples. The sample was fixed to the fixtures at both short sides. After measuring the sample’s length and width, the sample was loaded under tensile strain at a speed of 0.5 mm/min. The loaded force until rupture of the sample and the distance between both of the fixtures were measured automatically. The mechanical stress and tensile strain were calculated by using these data.

### Transmission electron microscopy (TEM)

Samples were minced into pieces with less than 0.1 mm^3^ total volume and fixed in 2.5% glutaraldehyde/PBS overnight. The samples were then processed and observed according to the standard TEM procedure.

### Protein Assay

Uterine tissue samples were washed briefly in PBS prior to wet weight measurement. Following this, samples were freeze-dried for a minimum of 8 hours using a vacuum freeze dryer (Eyela, Japan). The dried samples were immersed in a lysate buffer containing 446 µg/ml of papain, 5 mM cysteine-HCl and 5 mM EDTA-2 Na and incubated at 60°C for at minimum of 15 hours. The samples were further refined using a homogenizer and an ultrasonic cell disruptor. The end product of this process is referred as the “protein extracted sample”.

#### DNA assay

A commercially available DNA assay kit (Quant-iT PicoGreen dsDNA assay kit; Invitrogen, USA) was used to quantify the DNA contents. Each of the protein extracted samples was processed according to the standard protocol and analyzed with a fluorospectrophotometer at a wavelength of λ = 522 nm.

#### Hydroxyproline assay

Collagen protein contents of each sample were measured in terms of hydroxyproline content through the standard procedure [31]. Briefly, 50 µl of each protein extracted sample was mixed with 50 µl of 4 M NaOH and heated at 120°C for 30 minutes. Subsequently, 50 µl of 1.4 N citric acid and 250 µl of chloramine-T solution were added to the samples. After 20 minutes elapsed, 250 µl aldehyde-perchloric acid reagent was put into the solution and incubated at 70°C for 20 minutes. Each sample was analyzed with a fluorospectrophotometer at a wavelength of λ = 450 nm.

#### Elastin assay

A commercially available elastin assay kit (Fastin elastin assay kit; Biocolor, UK) was used for assessing elastin contents. 100 µl of each protein extracted sample was mixed with 50 µl of elastin precipitating reagent and incubated for 15 minutes. The sample was centrifuged at 10,000 g for 10 minutes and the precipitate was collected. 1 ml of dye reagent (TPPS: 5, 10, 15, 20-tetraphenyl-21, 23-porphine sulphonate) was added and mixed lightly for 90 minutes. The dye-bound elastin was collected by centrifugation at 12,000 g for 10 minutes, re-diluted by 250 µl of dye dissociation reagent. Each sample was analyzed with a fluorospectrophotometer at a wavelength of λ = 513 nm.

### Transplantation of samples

**Figure 1:**
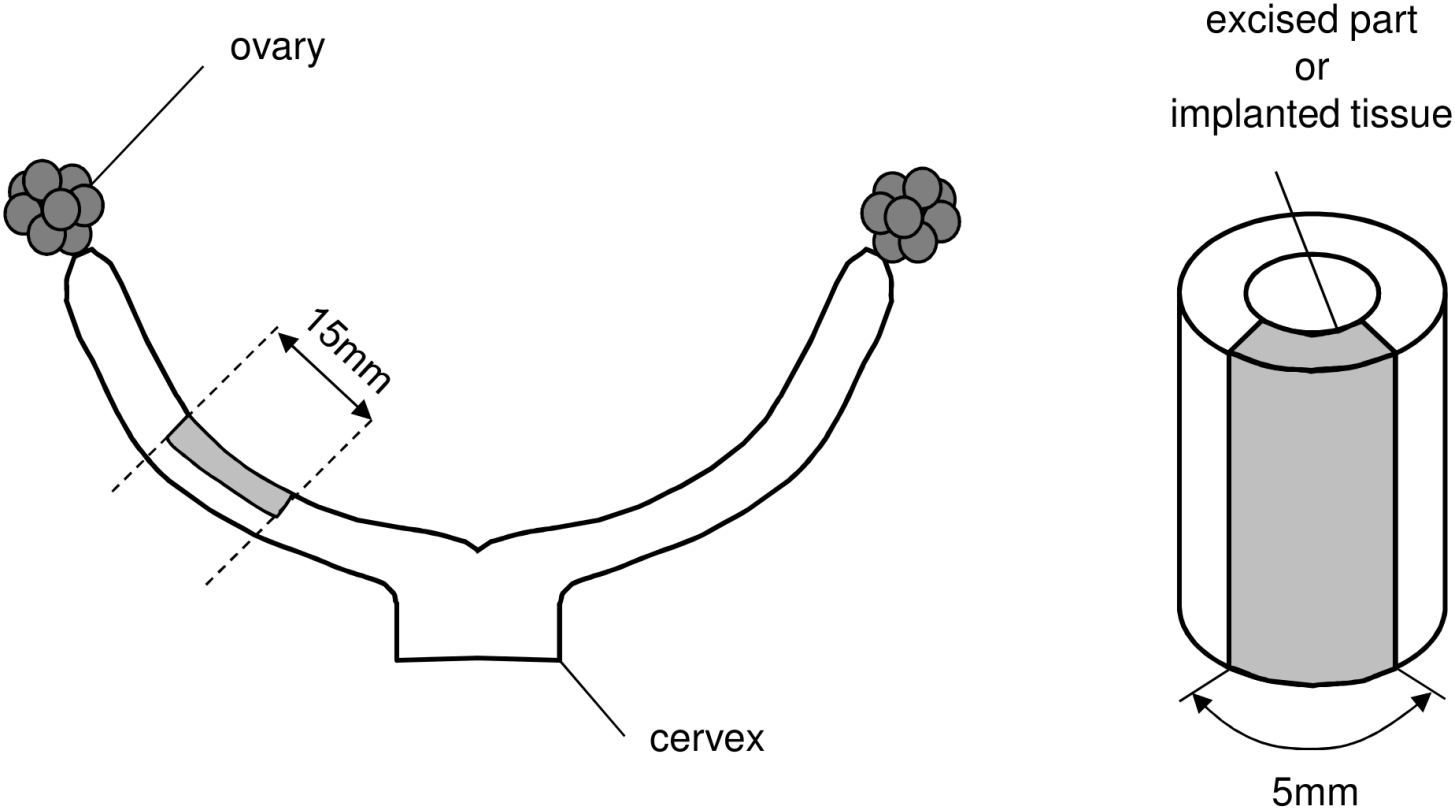
Illustrations of sample excision for decellularization and in-vivo study.

Twenty four uterine horns from SD rats (9 weeks old, female) at the proestrus or metestrus cycle were used for the experiments of the decellularized tissue transplantation. These horns were divided into three groups (n = 8, sham, SDS, and HHP). Sham operations refer to incising part of uterine horn (Fig. 1) and stitching it back without any modification. For SDS and HHP, part of the uterine horn was excised (Fig. 1) and replaced with SDS or HHP decellularized tissues extracted at the same point in the estrous cycle as the recipient. The decellularized tissue was sutured by a non-degradable polypropylene thread at 8 points. The points were on the sample’s corners and the some points on the sides of each sample. After suturing, the implanted part was covered with seprafilm (KAKEN, Japan), a postoperative film that prevents adhesion between the implanted part and surrounding fat tissues or other organs. The rats were kept for 1 month and sacrificed during the proestrus cycle. Implants were extracted and trimmed of connective tissue and fat. The samples were processed for histology, protein assay and mechanical testing.

### Evaluation of fertility after transplantation of decellularized tissues

Thirty two uterine horns from SD rats (9 weeks old female) at the proestrus cycle or metestrus cycle were used for the pregnancy test. These horns were divided into three groups: sham (n = 16), SDS (n = 8), and HHP (n = 8). Thirty days following the surgery (as previously described in section 2.7), the female rats were mated with fertile males to introduce pregnancy. Pregnancy was examined by the existence of sperm. Twenty one days after the confirmation of virginal sperms, the rats were sacrificed for the evaluation of pregnancy.

## Results

### Evaluation of decellularized tissue

**Figure 2:**
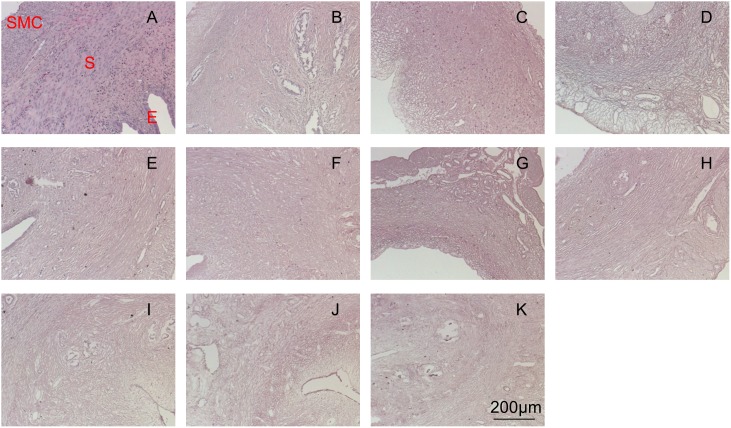
HE staining for native uterine tissue (A) and decellularized uterine tissue by 0.1%SDS detergent for 1 hour (B), 1%SDS detergent for 1 hour (C), 1%SDS detergent for 2 hours (D), HHP10-4 (E), HHP10-37 (F), HHP30-4 (G), HHP30-37 (H), 1%Triton-X detergent for 24 hours (I), 3%Triton-X detergent for 24 hours (J) and 3%Triton-X detergent for 48 hours (K). Legend in red refers to: E – epithelial layer, S – stroma layer and SMC – smooth muscle layer.

Evaluation of the decellularization efficiency was done by using hematoxylin and eosin staining. Native uterine tissue is presented in Fig. 2A as the control sample. Fig. 2B–D represents samples decellularized by SDS with various concentrations and time frames. The sample processed with 0.1% SDS for 1 hour (Fig. 2B) showed the most residual cells in the epithelial and stromal layers compared to the other samples. Treatment at 1% SDS for 1 hour (Fig. 2C) effectively removed cells in the smooth muscle and epithelial layers, but some cells still remained in the stromal layer. On the other hand, samples decellularized using 1% SDS for 2 hours (Fig. 2D) showed the best result with a thorough removal of the majority of smooth muscle cells and stromal cells.

HHP decellularized samples are shown in Fig. 2E–H. The samples were treated with different initial pressurization temperatures (10°C and 30°C) and washing temperatures (4°C and 37°C). These conditions are denoted by HHP ‘pressurization temperature’-‘washing temperature’ (e.g. HHP 10-4). HHP 10-4 (Fig. 2E), HHP 10-37 (Fig. 2F) and HHP 30-37 (Fig. 2H) samples exhibited similar results with a thorough removal of cells in the smooth muscle and stromal layers, but not in the epithelial layers. Contrary to these, HHP 30-4 (Fig. 2G) had full-thickness cell removal.

Triton-X decellularized samples are shown in Fig. 2I–K. Similar to the SDS group, decellularization was done by varying the concentration and duration of exposure. Samples decellularized by 1% Triton-X for 24 hours (Fig. 2I), 3% Triton-X for 24 hours (Fig. 2J) and 3% Triton-X for 48 hours (Fig. 2K) resulted in effective cell removal in the smooth muscle and stroma layers. However, epithelial cells were detected in all Triton-X group’s samples.

**Figure 3:**
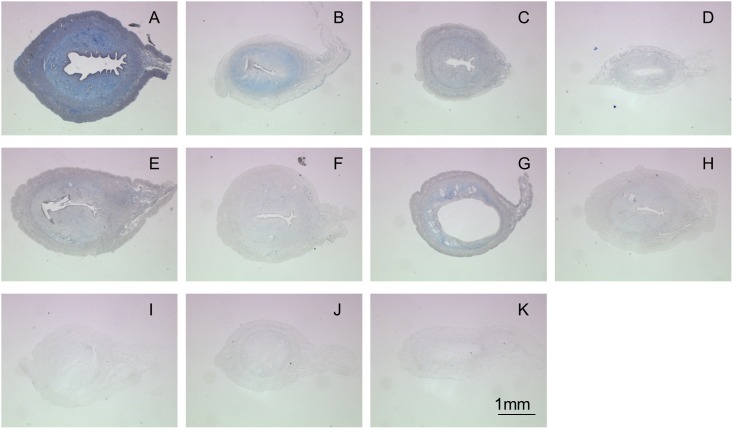
MT staining for native uterine tissue (A) and decellularized uterine tissue by 0.1%SDS detergent for 1 hour (B), 1%SDS detergent for 1 hour (C), 1%SDS detergent for 2 hours (D), HHP10-4 (E), HHP10-37 (F), HHP30-4 (G), HHP30-37 (H), 1%Triton-X detergent for 24 hours (I), 3%Triton-X detergent for 24 hours (J) and 3%Triton-X detergent for 48 hours (K).

Collagen protein helps maintain the elasticity of the uterine tissue and can be found in the form of fibers in the tissue. These fibers, like elastin, are susceptible to denaturation by external stresses or chemicals. Assessment of the collagen content in the ECM was done using Masson’s Trichrome staining. The intensity of Aniline Blue in decellularized matrices was compared to the native tissue (Fig. 3A) as a qualitative indicator of the reduction of collagen content. All of the decellularized uterine tissue samples (See Fig. 3B–3K) revealed a reduction in collagen content relative to the native tissue. The conditions in the SDS and HHP groups that had the highest residual collagen content after treatment are: 1% SDS for 1 hour, HHP 10-4 (Fig. 3E), and HHP 30-4 (Fig. 3G). In the Triton-X group (Fig. 3I–K) all conditions were shown to reduce the collagen content significantly compared to native tissue.

**Figure 4:**
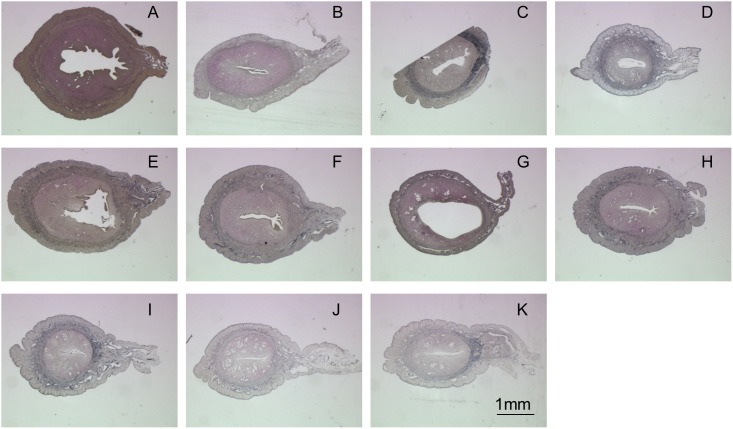
VVG staining for native uterine tissue (A) and decellularized uterine tissue by 0.1%SDS detergent for 1 hour (B), 1%SDS detergent for 1 hour (C), 1%SDS detergent for 2 hours (D), HHP10-4 (E), HHP10-37 (F), HHP30-4 (G), HHP30-37 (H), 1%Triton-X detergent for 24 hours (I), 3%Triton-X detergent for 24 hours (J) and 3%Triton-X detergent for 48 hours (K).

Verhoeff’s Van Gieson staining (Fig. 4) was used to detect the elastin content in the decellularized matrix. These fibers are stained dark purple and a qualitative analysis of the elastin content is done by comparing the intensity of the stain. Similar to the collagen content results, there was a reduction of elastic content across all samples relative to that of the native tissue. In the SDS group, 1% SDS for 1 hour (Fig. 4C) samples exhibited a higher elastin content compared to the other two conditions. In the HHP group, HHP 10-4 (Fig. 4E) and HHP 30-4 (Fig. 4G) showed a similar elastin content, and were stained more intensely than the other two samples in the HHP group (Fig. 4F and 4H). Within the Triton-X group (Fig. 4I to 4K), treatment with 1% Triton-X for 24 hours (Fig. 4I) had the highest elastin content; however, the staining was less intense compared to SDS and HHP group.

For further evaluation, the best decellularization method from each category (excluding Triton-X which is not suitable for uterine tissue) were picked, namely, 1% SDS for 1 hour (Fig. 2C, 3C and 4C) and HHP 30-4 (Fig. 2G, 3G and 4G).

**Figure 5:**
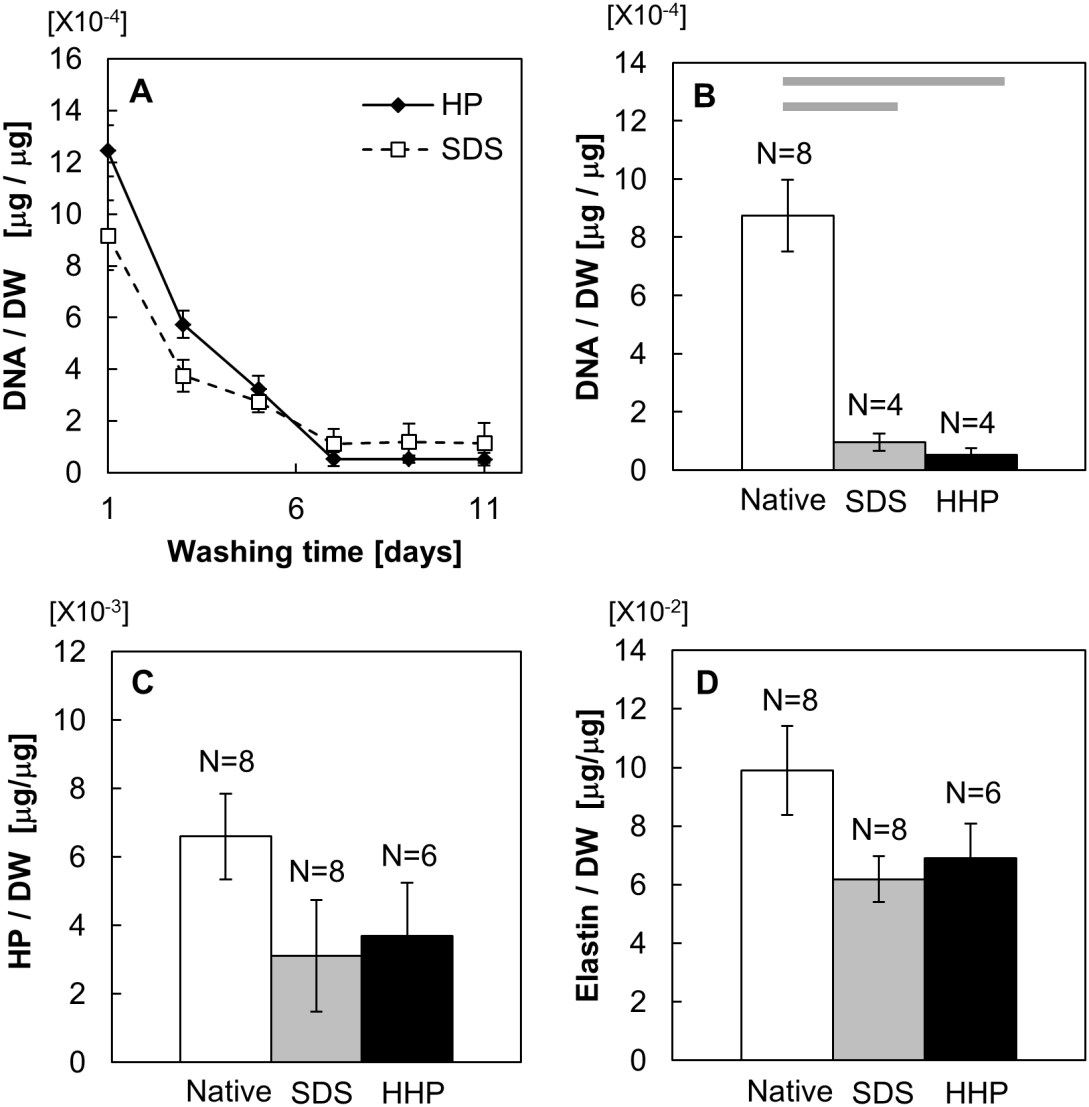
Quantification of protein contents of native and decellularized uterine tissue in terms of the temporal change of the DNA amount (A), contents of DNA (B), hydroxyproline (C) and elastin (D). Data are presented as mean ± SE. Bars indicate p<0.05.

The DNA contents of the decellularized tissues were quantified to confirm the effectiveness of the cell removal methods. Fig. 5A shows that the SDS and HHP treatments reduced the DNA contents in uterine tissues with time. The remaining DNA contents in the tissues reached a plateau at 7 days. At 11 days washing, there was no statistical difference in the DNA contents in the tissues between SDS and HHP treatments. As shown in Fig. 5B, the remaining DNA contents at 7 days washing for SDS (0.96±0.30×10^−4 ^µg/µg) and HHP (0.53±0.22×10^−4 ^µg/µg) tissues were significantly lower than that of the native tissues (8.75±0.12×10^−4 ^µg/µg), with a lower DNA content in HHP tissues compared to SDS tissues. At 7 days washing, the hydroxyproline (HP) content of decellularized tissues was also measured. In Fig. 5C, the HP content per dry weight for the SDS and HHP group (SDS: 3.11±1.61×10^−1 ^µg/µg, HHP: 3.69±1.63×10^−1 ^µg/µg) were 52% and 44% lower than the native tissue (6.60±1.25×10^−1 ^µg/µg). Contrary to this, the elastin contents of SDS (6.07±0.96×10^−2 ^µg/µg) and HHP (6.91±1.25×10^−2 ^µg/µg) were 39% and 30% lower, respectively, than the one of native tissue (9.90±1.53×10^−2 ^µg/µg) (Fig. 5D).

**Figure 6:**
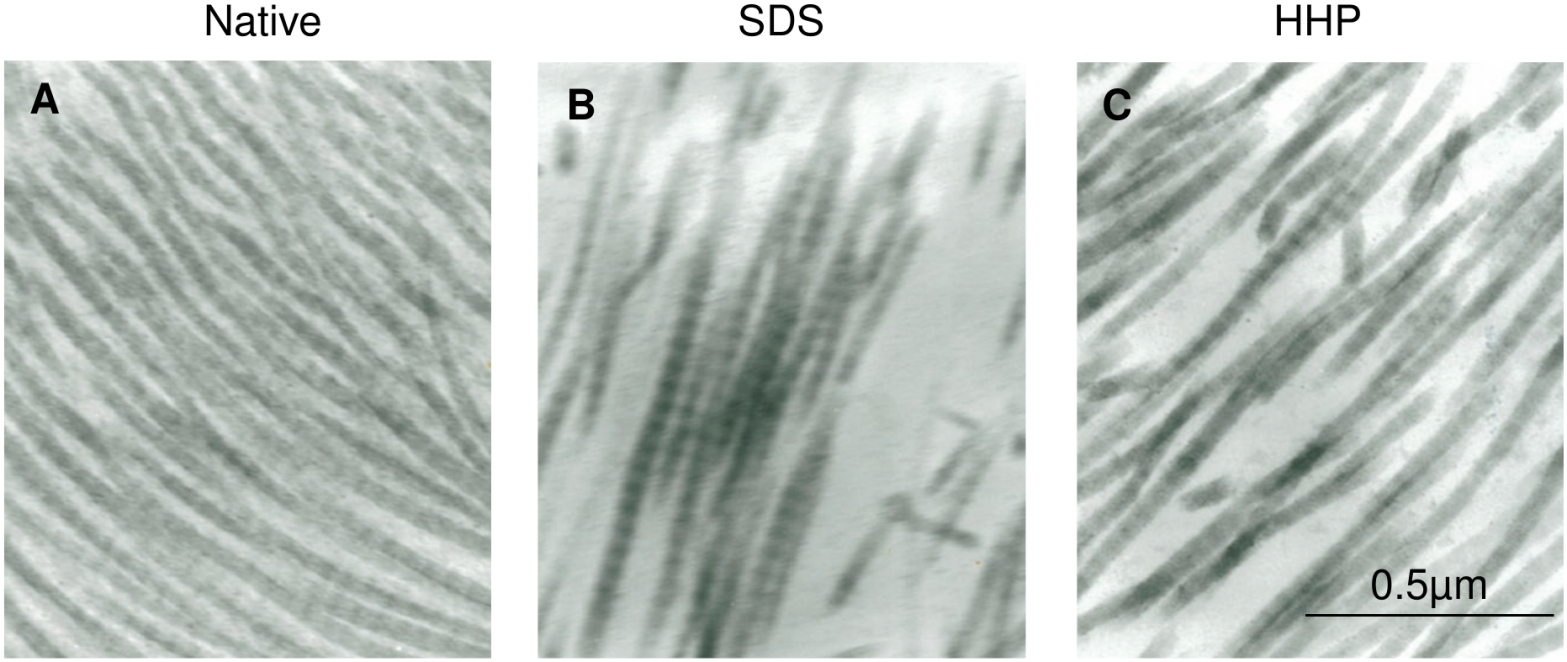
TEM picture of collagen fibers in native uterine tissue (A), SDS 1% for 1 hour (B) and High hydrostatic pressure 30-4 (C).

The collagen density and structure of the tissues was visualized through TEM (Fig. 6A). Fig. 6B and 6C show the collagen fibers of decellularized matrices by SDS and HHP, respectively. In both decellularized samples, a reduction in the number of fibers was observed. Nonetheless, the structure of the residual collagen fibers was preserved.

**Figure 7:**
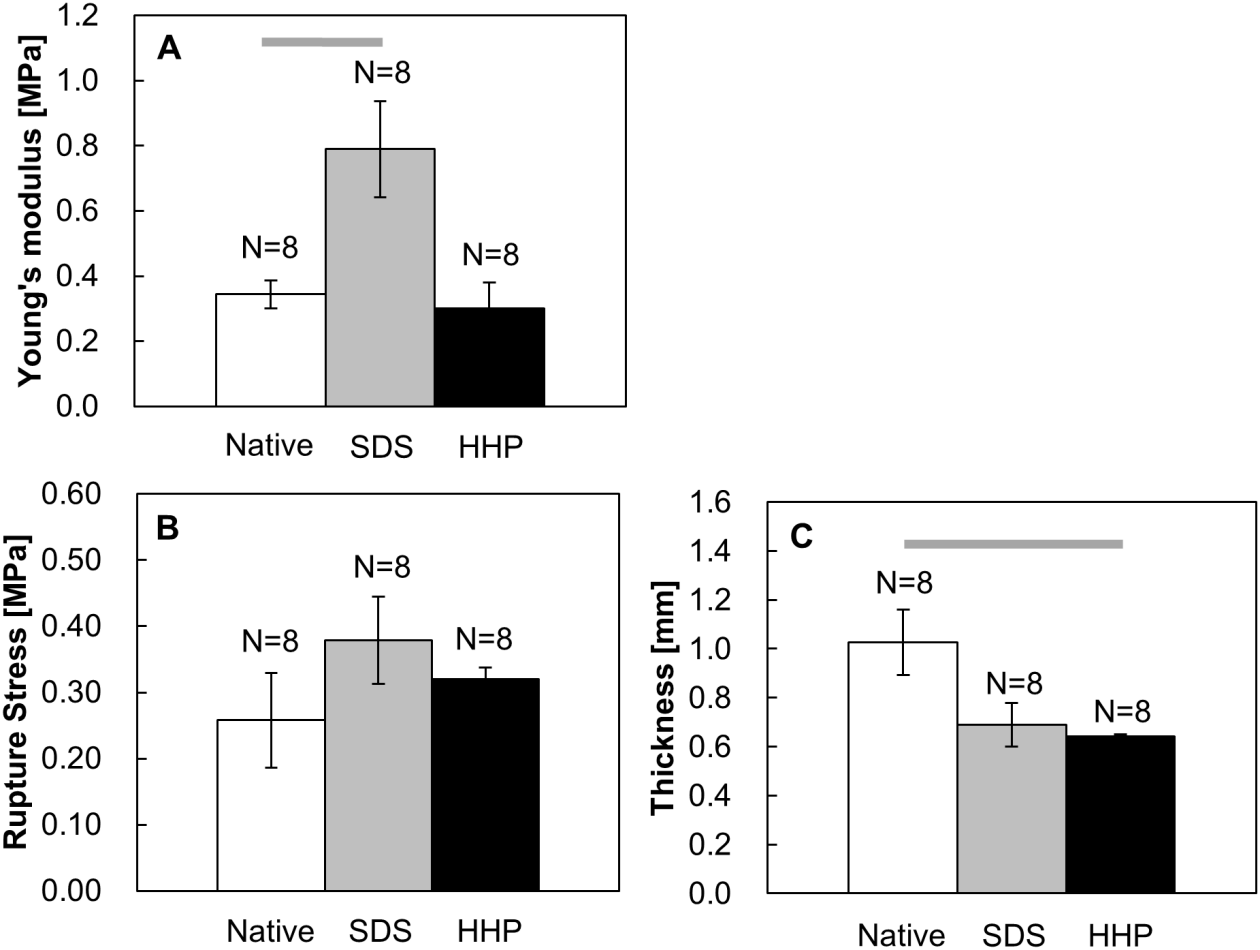
Mechanical properties of native and decellularized uterine tissues. Young’s modulus (A), rupture stress (B) and thickness (C). Data are presented as mean ± SE. Bars indicate p<0.05.

Next, the thickness and mechanical properties of the decellularized uterine tissues were evaluated as shown in Fig. 7. The Young’s modulus of SDS-treated samples (0.688±0.131 MPa) was 2.00 and 2.29 times higher than those of the native and HHP samples (native: 0.344±0.043 MPa, HHP: 0.300±0.079 MPa, Fig. 7A). There was a statistically significant increase in the Young’s Modulus of SDS-treated tissues compared to the native tissues (p<0.05). Similarly, the rupture strength of the SDS group (0.379±0.065 MPa) was 1.45 and 1.18 times higher than that of the native tissue (0.258±0.071 MPa) and HHP-treated (0.320±0.017 MPa) samples (Fig. 7B). However, there was no significant difference between the SDS group and native tissues. HHP- treated samples showed similar levels in Young’s modulus and rupture strength to native tissues. There were no statistical differences in the mechanical strength between the HHP group and native tissues. The thickness of SDS- (0.66±0.10 mm) and HHP- (0.64±0.01 mm) decellularized tissues decreased 36% and 38%, respectively, in comparison to the native tissue (1.03±0.13 mm) (Fig. 7C).

### In vivo evaluation

**Figure 8:**
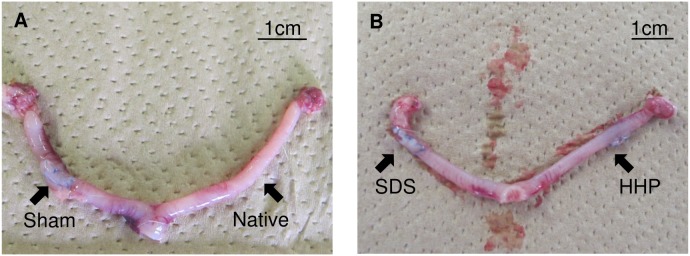
Photograph of native uterus (A) and reconstructed uterus (B) with excision sites as labelled.

In vivo transplantation experiments are necessary to determine the regenerative capability of decellularized tissues with native tissues. Thirty days after transplantation, uterine tissues were extracted from the rats and the gross evaluation of the tissue is presented in Fig. 8. Comparison between the native (Fig. 8A-Right), sham (Fig. 8A-Left), HHP-(Fig. 8B-Right) and SDS-(Fig. 8B-Left) treated tissues show no noticeable differences. The implants seemed to fully integrate with the surrounding native tissue without any inflammation.

**Figure 9:**
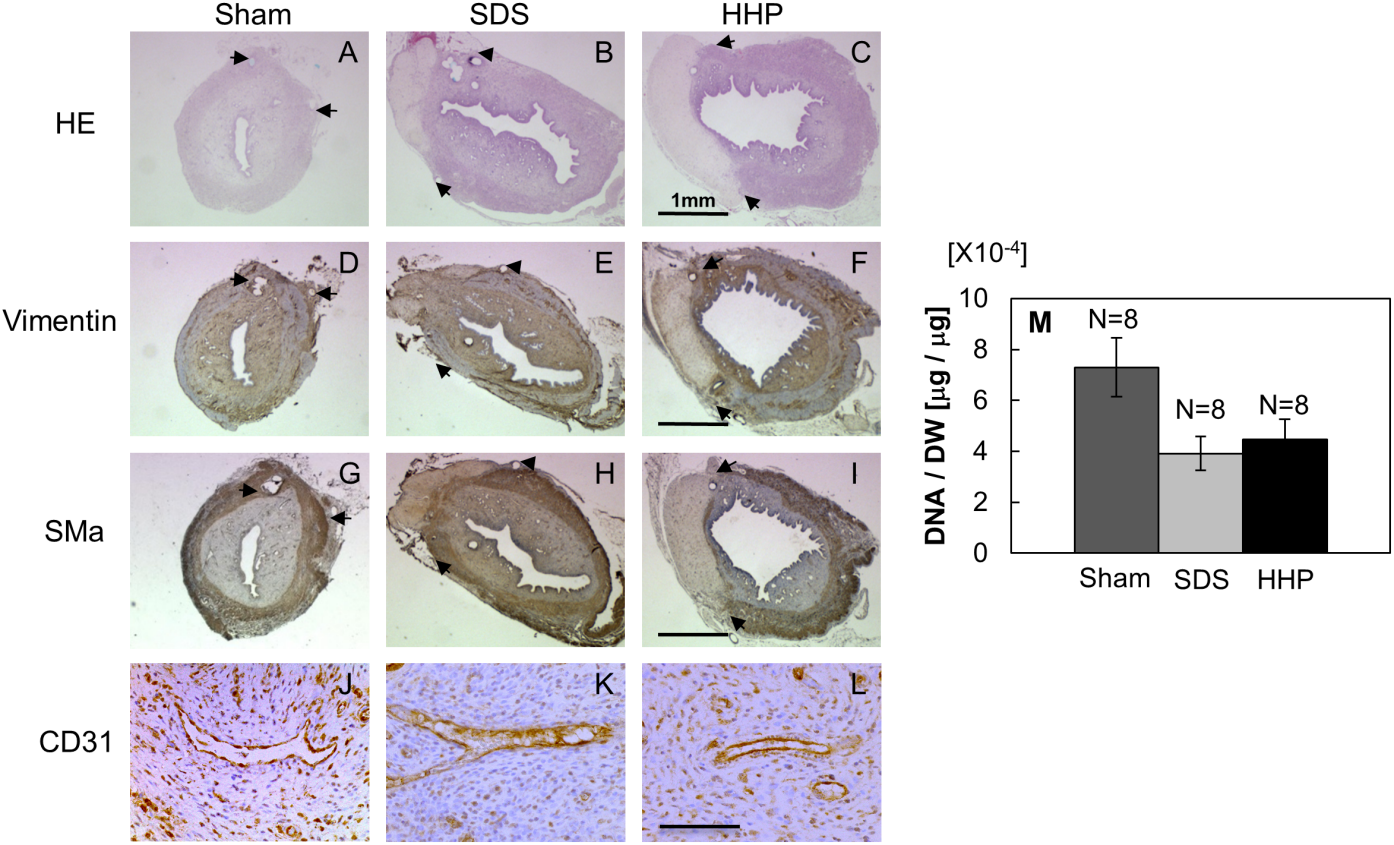
Qualitative observation of reconstructed uterus for tissue regeneration at days 30 (H&E, vimentin and α-SMA) and blood vessel (CD31), respectively, in sham (A, D, G and J), SDS 1% for 1 hour (B, E, H and K) and HHP 30-4 (C, F, I and L), and quantitative analysis of DNA contents (M). Data are presented as mean ± SE. Arrows in the picture indicate the interface of implant and native tissue.

At day 30, decellularized uterine tissues using HHP and SDS methods showed tissue regeneration and epithelial cell migration into the implanted area (Fig. 9B, C). In both reconstructed uterine tissues, regeneration of stromal layers underneath the implant area was detected with vimentin staining (Fig. 9E, F). Similarly, the regeneration of the smooth muscle layer was observed for HHP- and SDS-decellularized tissues (Fig. 9H, I). CD31 staining revealed blood vessels present in the reconstructed uterine tissues underneath both decellularized tissues (Fig. 9K, L).

From the quantitative analysis, DNA contents per dry weight in the SDS (3.91±0.72×10^−4 ^µg/µg) and HHP (4.53±0.84×10^−4 ^µg/µg) groups were 46% and 38% lower than those in the sham group (7.30±1.23×10^−4 ^µg/µg, Fig. 9M).

**Figure 10:**
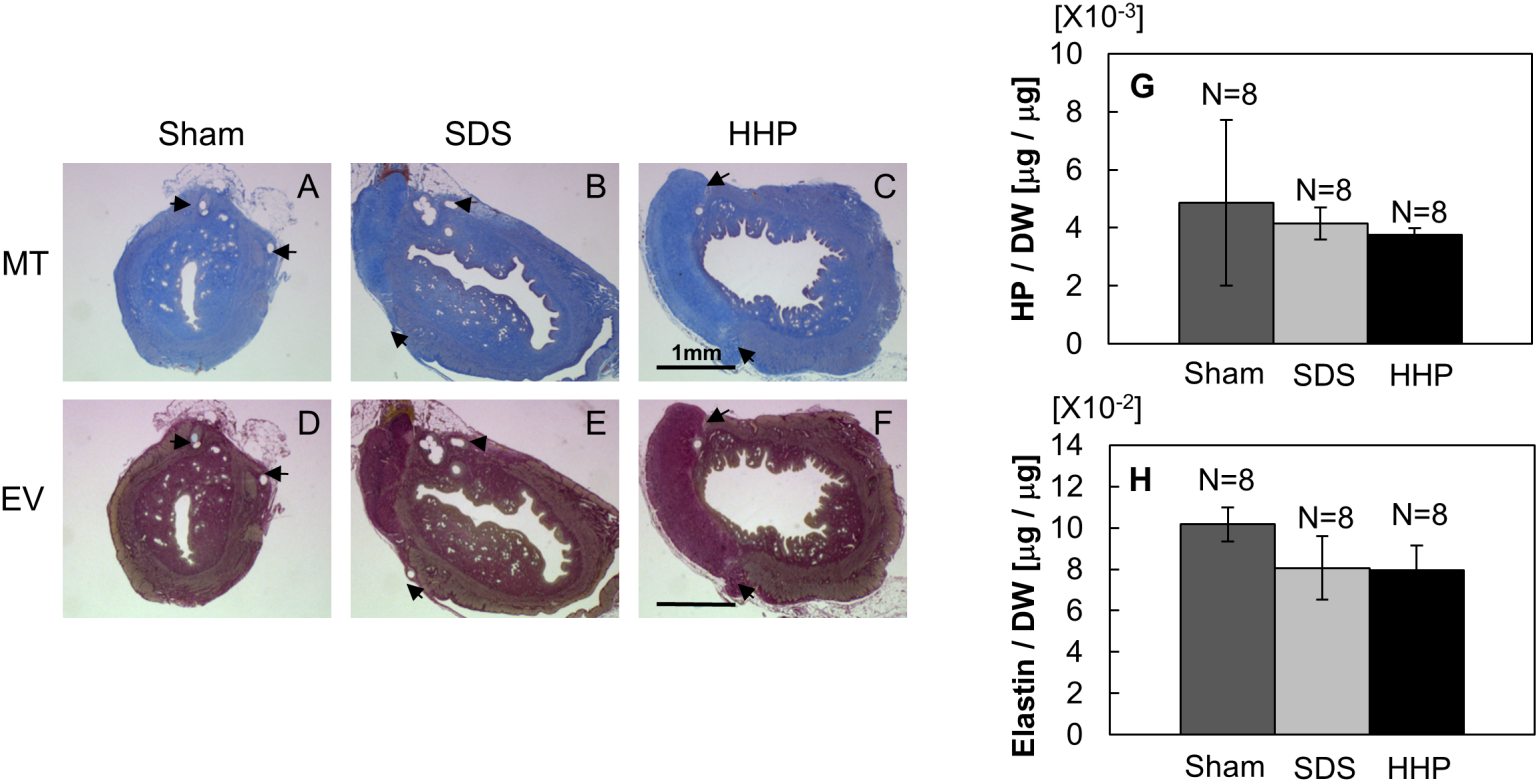
Qualitative observation of reconstructed uterus at days 30 for collagen (MT) and elastin (VVG), respectively, in sham (A and D), SDS 1% for 1 hour (B and E) and HHP 30-4 (C and F); and quantitative analysis of hydroxyproline (G) and elastin (H). Arrows in the picture indicate the interface of implant and native tissue. Data are presented as mean ± SE.

Masson’s Trichrome staining was used to detect the recovery of the collagen proteins in the implant that were denatured during decellularization. In both SDS (Fig. 10B) and HHP (Fig. 10C) samples, the aniline blue intensity was comparable to the sham group (Fig. 10A) signifying the growth of collagen protein from the regenerated tissue into the implant. Verhoeff’s Van Gieson staining was used to observe the restoration of elastin protein in the implant. Similar to the Masson’s Trichrome staining results, the elastin protein in SDS (Fig. 10E) and HHP (Fig. 10F) groups increased and are comparable to the sham group (Fig. 10D) 30 days after transplantation. The quantitative results of HP and elastin contents are presented in Fig. 10G and 10H, respectively. The HP contents of SDS (4.14±0.56×10^−3 ^µg/µg) and HHP (3.76±0.23×10^−3 ^µg/µg) were at the same level as the sham groups (4.86±2.85×10^−3 ^µg/µg, Fig. 10G). On the other hand, there was a slight decrease in the elastin contents of SDS (0.80±0.12×10^−1 ^µg/µg) and HHP (0.81±0.15×10^−1 ^µg/µg) samples compared to the sham (1.02±0.08×10^−1 ^µg/µg) samples (Fig. 10H); although, with both the HP and elastin quantitative results, there are no significant differences between all samples.

**Figure 11:**
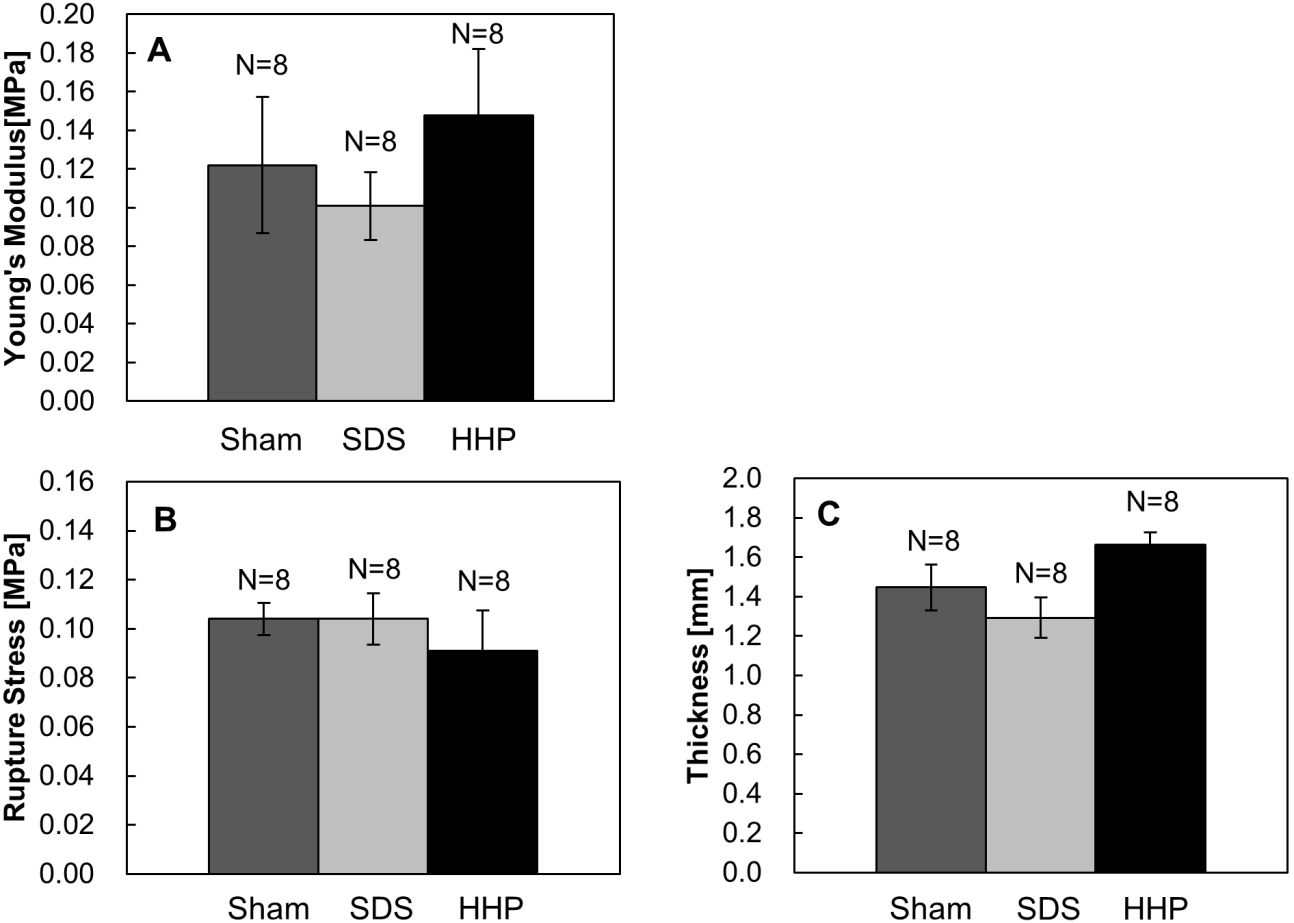
Mechanical properties of reconstructed uterus at day 30. Young’s modulus (A), rupture stress (B) and thickness (C). Data are presented as mean ± SE.

The mechanical properties and thickness of the implanted tissues are shown in Fig. 11A–C. The Young’s modulus of the decellularized tissues (SDS: 0.101±0.018 MPa, HHP: 0.148±0.034 MPa) were not significantly different from the sham (0.122±0.035 MPa) (Fig. 11A). Similarly, regarding of the rupture strength, large differences were not detected between the sham (0.098±0.007 MPa), SDS (0.104±0.007 MPa), and HHP (0.086±0.014 MPa) tissues. Also, in comparison to the thickness of sham (1.38±0.12 mm), SDS (1.29±0.07 mm) and HHP (1.70±0.06 mm) was at the same level.

**Figure 12:**
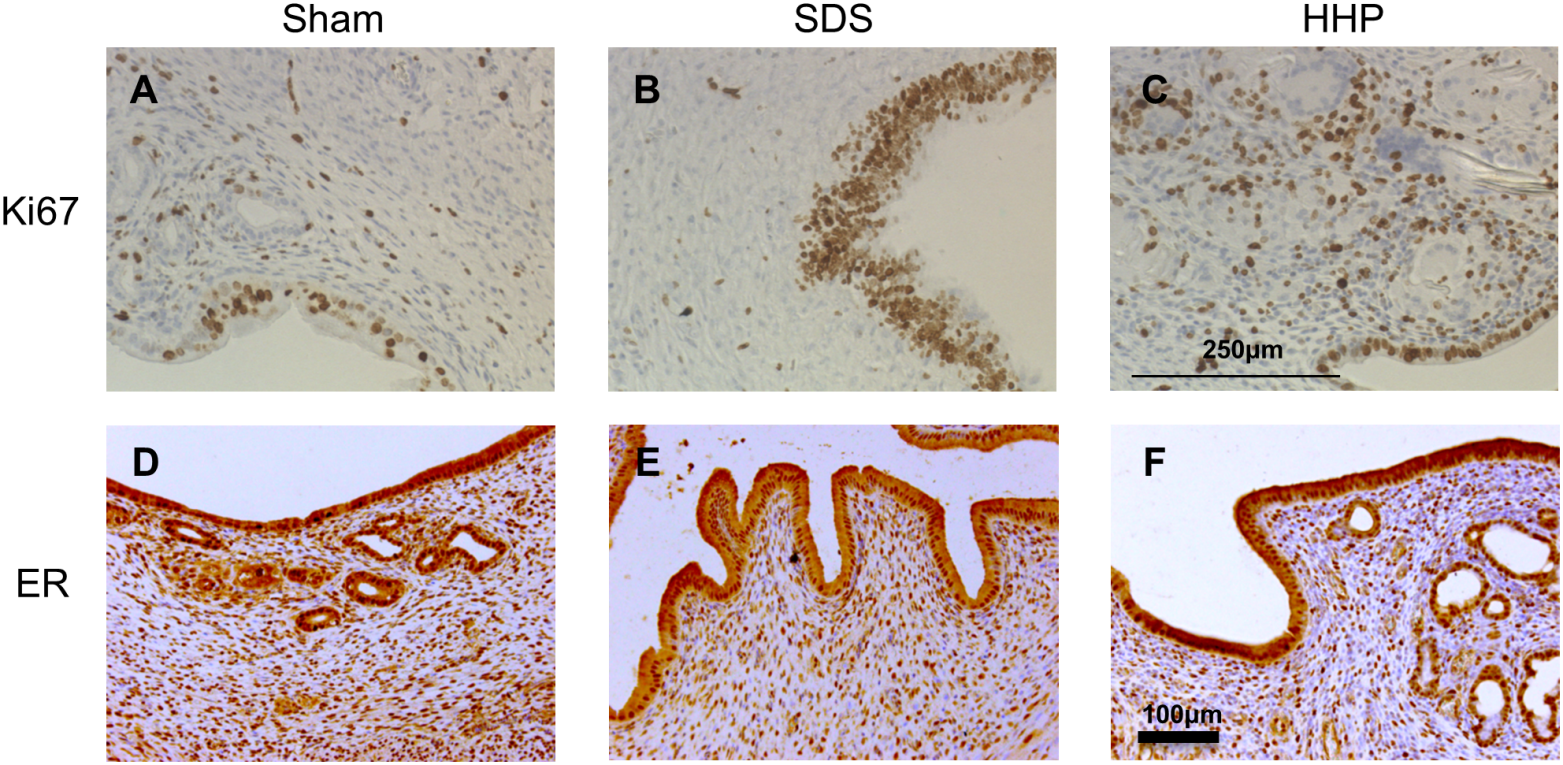
Immunostaining of Ki67 (A–C) and estrogen receptor (ER, D–F), respectively, in sham (A and D), SDS 1% for 1 hour (B and E) and HHP 30-4 (C and F) at days 30. Arrows in the picture indicate the interface of implant and native tissue.

Responsiveness of the implant to ovarian hormone was ascertained by using Ki67, a marker for proliferating cells. Both SDS (Fig. 12B) and HHP (Fig. 12C) reconstructed uterine tissues had a positive Ki67 staining in the luminal epithelium and negative staining in the stroma, which is the normal proliferation pattern in the proestrus uterus. A higher number of proliferative cells in stromal layer were detected as brown colored particles in HHP group. Evaluation of the ER expression (dark orange staining), which reveals the responsiveness to the ovarian hormone for pregnancy in the reconstructed tissues, showed, as expected, ER-positive immunostaining both in the luminal epithelium and the stromal cells for both samples from the SDS (Fig. 12E) and HHP (Fig. 12F) group. Positive staining of the glandular epithelium in HHP (Fig. 12E) and SDS (data not shown) samples was exhibited the same as sham samples.

**Figure 13:**
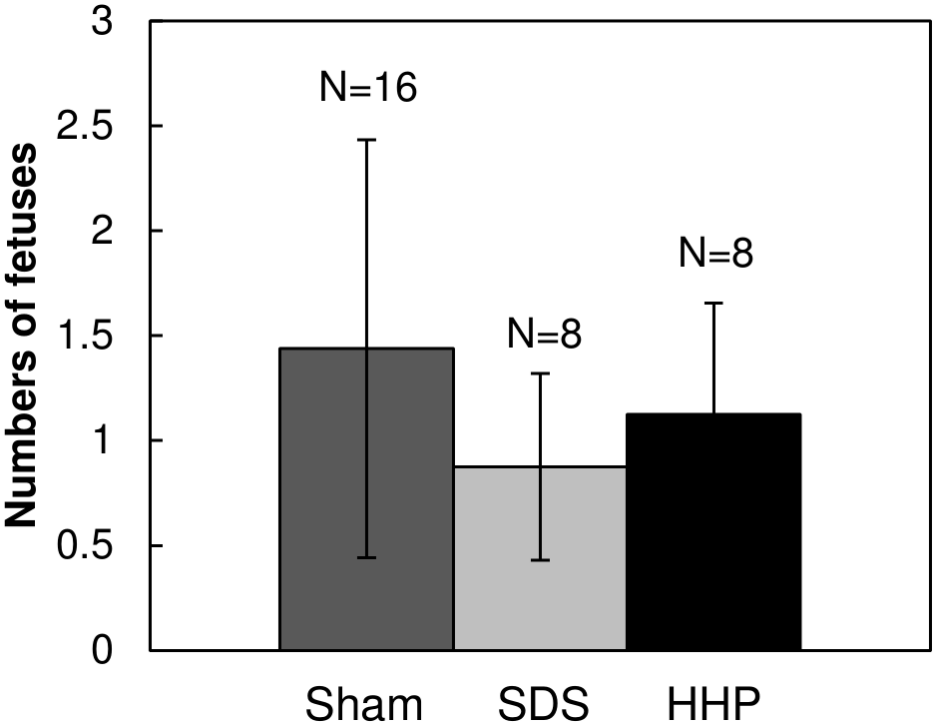
Numbers of fetuses in the reconstructed uterine horns on day 21 of pregnancy. Data are presented as mean ± SE.

Thirty days after the transplantation of decellularized tissues, we evaluated fertility of the rats on day 21 of pregnancy. As presented in Fig. 13, the numbers of fetuses were comparable among the sham, SDS and HHP groups (1.44±0.99, 0.88±0.43 and 1.13±0.58, respectively).

## Discussion

Decellularized matrices have been used for the treatment of several organs over biomaterials due to its practicality and lower immune reactivity. To our knowledge, we are the first to report on decellularized matrices for uterine tissue engineering. The optimization of conditions for decellularization is essential, so various methods for decellularization were evaluated for both the efficiency of cell removal and the preservation of the physical, chemical and biological integrity of rat uterine tissues. In this research, three kinds of decellularization methods were selected: SDS due to its efficiency in removing cells from dense material, HHP for its capability to preserve collagen and elastin, as well as, Triton-X because of its ability to decellularized thick tissues.

From the histological results, decellularization using SDS was the most effective in removing cells within the smooth muscle layers. SDS decellularizes the tissue through diffusion from the smooth muscle layer towards the epithelial layer, so the application time used in this study may have been too short for the detergent to reach the epithelial layer. Although prolonging the decellularization time results in greater cell removal (Fig. 2C vs. 2D), the detergent would also denature or reduced collagen and elastin proteins in the tissue in the process (Fig. 3C vs. 3D and 4C vs. 4D).

Similar to SDS, Triton-X also utilizes diffusion for decellularization. At the conditions used in this study, Triton-X did not manage to penetrate the uterine tissue all the way to the epithelial layers. Compared to SDS at a similar cell removal efficiency, Triton-X required more time (Fig. 2D vs. 2I). Moreover, Triton-X severely denatured or reduced the collagen and elastin protein within the matrix (Fig. 3D vs. 3I and 4D vs. 4I). Thus, for uterine tissues, Triton-X was deemed as unsuitable.

In HHP, decellularization is achieved by applying a high pressure to the tissue to disrupt the cell membrane. Thus, in principle, during the decellularization process, cells can effectively be removed from all layers without denaturing or reducing the proteins. However, as previously studied by Funamoto et al. [29], the pressure-temperature relationship during HHP is an important factor. It directly determines whether the tissue reaches the freezing zone where water in the tissue is converted to ice. Ice crystal formation can cause modification to tissue structure in the form of scissions of the collagen fibers. The freezing zone can be avoided by using an onset temperature of 30°C, as shown by the higher collagen fiber content in Fig. 3G compared to Fig. 3E, which used an onset temperature of 10°C. A similar result of higher collagen content in conjunction with a higher onset temperature was seen by a more intense VVG staining of the HHP 30-4 sample (Fig. 4G) when compared to the HHP 10-4 sample (Fig. 4E). The reduction of the collagen and elastin contents within the tissue processed at 30°C (Fig. 3G, H and Fig. 4G, H) are the result of the enzyme contained in the washing process. Since the enzyme activity is higher at 37°C than 4°C, a lower collagen and elastin content was detected in samples washed at 37°C (Fig. 3H and 4H). A similar washing procedure was also used for samples with a 10°C onset temperature (Fig. 3E–F and Fig. 4E–F), and a collagen and elastin reduction due to the enzyme activity was also observed in samples washed at 37°C (Fig. 3F and Fig. 4F). Based on these results, for the purpose of uterine tissue decellularization, HHP (specifically HHP 30-4) offers a higher cell removal efficiency and ECM preservation.

Additionally, protein quantification, TEM and mechanical tests were used to objectively compare the residual content between different decellularization methods. The optimal conditions of each decellularization method (excluding Triton-X) were determined to be: SDS 1% for 1 hour and HHP 30-4.

Comparison of the residual collagen content (Fig. 5C, Fig. 6) showed that HHP didn’t alter the collagen fibers significantly, whereas SDS destroyed the collagen fibers. Further investigation of the collagen structure using TEM elucidated that even though the denaturation of the collagen protein reduced the number of fibers, the structure of the residual collagen fibers was preserved. These results indicate that there was a minimal overall change in the structural properties during both decellularization processes. Residual elastin content (Fig. 5D) showed that elastin fibers, unlike collagen, were susceptible to both pressure and chemical.

The values of the Young’s Modulus in SDS samples were significantly higher than the native and HHP samples before transplantation, as shown in Fig. 7A and B. Since SDS is a detergent, it is possible that the SDS treatment resulted in the denaturation of collagen fibers. From the TEM analysis (Fig. 6A–C), the number of collagen fibers seemed to be reduced in the SDS samples, but collagen structure between SDS and HHP samples did not differ. Thus, as a possible cause, it was considered that the change in higher-ordered structure of collagen fibers such as collagen-collagen interactions induced denaturation which influenced in the stiffness of SDS samples.

DNA quantification (Fig. 5A) showed that while both decellularization methods were effective in removing cells, it was impossible to completely remove the DNA content from the tissue. To remove the DNA completely, harsher conditions for decellularization, such as a longer treatment time with HHP or SDS, and higher concentrations of SDS, should be considered. Nonetheless, due to the required collagen and elastin fibers in the matrix in order to maintain the mechanical properties of the tissue, more extreme conditions were not deemed appropriate. Despite the residual DNA content, the in vivo study of the decellularized tissue proves that the amount of DNA removed was sufficient in preventing an immune reaction.

Gross examination of the in vivo study showed the integration of decellularized tissue with the native tissue within 30 days. No obstruction was observed at the implant/native tissue anastomoses as opposed to a study by Jonkman et al. [32] in which the artery were occluded by blood clot due to failure of vascularization into scaffold. Patency of the reconstructed uterus was similar to native tissue proving decellularized tissue to be superior to porcine SIS graft [33] where samples larger than 1 mm were found to be twisted due to lack of mechanical strength. Thickness and DNA content of the SDS and HHP group prior to (Fig. 7C and 5B) and after (Fig. 11C and 9M) the in vivo study showed an increase, signifying the existence of tissue regeneration. From H&E staining (Fig. 9B, C), it was evident that the mode of uterine reconstruction for the SDS group was re-growth of tissue from native uterine tissue (tissue regeneration) underneath the decellularized matrix. However, in the HHP group, it was a combination of cell migration and tissue regeneration. Qualitatively, thicker tissue regeneration underneath the decellularized tissue was observed in the SDS group. The faster regeneration in SDS group is hypothesized to be the result of mechanical difference between the decellularized and native tissue, which creates a mechanical stimulus for cell proliferation and growth. Conversely, due to similarities in both the protein content and the mechanical properties of the HHP-decellularized and native tissue, the cells chose to migrate into the implant. However, due to the lack of microvasculature to supply nutrition, cell migration was limited to the area near the native tissue. In SDS, cell migration was not observed, which was potentially caused by the lack of protein for cell-matrix signaling and cell binding.

At day 30, both the stromal and smooth muscle layer of the SDS reconstructed uterus showed a similar thickness to the sham group, which was representative of native tissue. In contrast, a similar study conducted by Li et al. [18] using a collagen scaffold showed that the regeneration of the smooth muscle layer to a thickness similar to sham group could only be achieved after 90 days of transplantation. Based on this, SDS decellularized tissue seems to encourage a higher regeneration rate compared to a collagen scaffold.

As mentioned previously, the collagen and elastin content is closely related to mechanical strength. Through the recovery of the collagen and elastin contents, the mechanical properties of the decellularized tissues became comparable to the sham group. However, the mechanical properties of the sham were lower than the native tissue (Fig. 7A, B and Fig. 11A, B), which may be due to imperfect surgical procedure causing tissue adhesion. During the tissue extraction, there was a noticeable increase in adipose tissue surrounding the uterine tissues, especially at the implant region. Most of the adipose tissue was trimmed off, but complete removal was not possible. As a result, the adipose tissue contributed to a higher overall implant thickness (Fig. 11C), which was supported by the fact that the thickness of the implanted tissues including the sham was higher than the one of native tissue (Fig. 7C and 11C). With increased thickness, the cross-sectional area of the sample would be increased and the Young’s modulus and rupture stress would be reduced. Additionally, the non-degradable polypropylene sutures in the implanted tissues act as a point defect, which will cause a decrease in the resistance to loads during tensile tests.

Responsiveness of the reconstructed uterus to the ovarian hormone was evaluated by immunostaining. It is known that estrogen governs the cell proliferation in the proestrus uterus. Therefore, Ki67 positive staining suggests that the responsiveness to estrogen is normal in the reconstructed tissues. Since estrogen acts via ER and governs the estrus cycle and uterine cell composition for pregnancy, ER positive staining indicates that SDS- and HHP-decellularized matrices provide the reconstructed uterus with normal hormone responsiveness and functions.

To confirm the functionality of reconstructed uterus, we examined the fertility of the female rats with transplantation. Based on the numbers of fetuses on day 21 pregnancy, both SDS and HHP groups showed similar fertility to the sham group, suggesting the successful reconstruction of the uterus by both decellularization methods.

## Conclusions

In this study, we focused on decellularized tissues for segmental uterine reconstruction; however, these methods are also applicable for larger samples such as the whole uterus. According to the biochemical and mechanical evaluation, decellularization using solutions such as SDS and Triton-X is highly dependent on various diffusion factors and their interaction with the sample; thus, the optimization of the solution’s concentration and application time (depending on the sample’s thickness, pore size, etc) is necessary to avoid under or over decellularization. Due to the variation in organ size from one person to another, the variables for chemical decellularization must be individually optimized for each case. In contrast, in HHP decellularization there is no need to customize the decellularization conditions as they are independent of the sample’s size and structure. Moreover, decellularization by HHP causes minimal protein denaturation in contrast to SDS and Triton-X, resulting in the superior preservation of the ECM content. In addition, residual SDS and Triton-X within the tissue can introduce a toxic response from the host. This problem can be avoided in HHP since no harmful chemicals are involved in the decellularization process. Therefore, HHP decellularization is the better option as a scaffold in uterine regeneration.

Immunohistochemical staining of the in vivo study showed that the regenerated tissues were positive for Ki67 and ER in both the SDS and HHP group. Specifically in the HHP group, we found cell migration near the native/implant anastomoses, showing the possibility for a novel reconstruction method.

Functionality in the form of hormone responsiveness and mechanical properties revealed the potential of reconstructed uteruses to behave as a native uterus. In the fertility tests, we found successful pregnancy in both the SDS and HHP groups similar to the sham model in term of number of fetuses. These findings indicate the possibility of a regular pregnancy and childbearing in the uteruses reconstructed by decellularized tissues.

All of the above results support our hypothesis that decellularized tissues could be used as a novel scaffold for clinical applications in uterine tissue engineering.

The use of decellularized tissue in whole uterine decellularization could potentially lead to a tissue engineered uterus with a low immune response, which could be an alternative to uterine transplantation by a donor. This would be beneficial for women who have past history of hysterectomy or uterine malformation and want to experience the childbearing process.
